# Is hypertension an extra‐intestinal manifestation of inflammatory bowel disease?

**DOI:** 10.1002/ueg2.12359

**Published:** 2023-01-19

**Authors:** Johan Burisch

**Affiliations:** ^1^ Gastrounit, Medical Division Copenhagen University Hospital – Amager and Hvidovre Hvidovre Denmark; ^2^ Copenhagen Center for Inflammatory Bowel Disease in Children Adolescents and Adults Copenhagen University Hospital – Amager and Hvidovre Hvidovre Denmark

Inflammatory bowel diseases (IBDs) are systemic diseases, that can manifest far beyond the gastro‐intestinal tract. Extra‐intestinal conditions affect up to half all patients with IBD and include a broad range of chronic diseases that share the pathophysiology with IBD, are consequences of intestinal disease activity, or complications induced by IBD treatments.^1^ This adds to the complexity of—ideally multidisciplinary ‐ IBD care^2^ as well as to the potential burden for the patient and to the health care system. Patients and physicians are required to be vigilant in the surveillance of extra‐intestinal manifestations and complications of IBD.

Recent studies have expanded the scope of conditions associated with IBD. As it the case for other immune‐mediated diseases, patients with IBD are at a increased risk of cardiovascular disease at a level similar to patients with type 2 diabetes.^3^ Furthermore, studies suggest that the inflammatory process per se, rather than any individual condition, is the risk factor^4^, ^5^ and that this increased risk may be driven by the contribution of chronic systemic inflammation to atherosclerosis.^6^ Hypertension is a major modifiable cardiovascular disease risk factor that a common manifestation of atherosclerosis. Hypertension is prevalent in patients with immune‐mediated disease such as rheumatoid arthritis and psoriasis^7^, ^8^ and has also been associated with systemic inflammation.^9^ However, data on the prevalence of hypertension in patients with IBD are sparse.

In this issue of *United European Gastroenterology Journal*, He and colleagues present interesting new insights in the prevalence of hypertension in patients with IBD.^10^ From the UK Biobank cohort—a well‐described cohort of more than 500,000 individuals from the United Kingdom ‐ the authors identified over 280,000 individuals with no history of hypertension, cardiovascular disease, diabetes, or stroke at inclusion and followed them for a median of 8 years. Of those, 2376 (0.8% of the total cohort) had IBD at inclusion and the authors found that compared to non‐IBD patients, patients with ulcerative colitis had a greater risk of developing hypertension (Hazard ratio 1.30, 95% confidence interval: 1.11–1.52) during follow‐up, while patients with Crohn's disease had not. In addition to having a diagnosis of ulcerative colitis, use of steroids as well as immunomodulators were identified as risk factors for hypertension with comparable hazard ratios.

The growing understanding of the impact of systemic inflammation on cardiovascular and thromboembolic complications to IBD, an ageing IBD‐population,^11^ as well as the introduction of new treatments with potential cardiac adverse events^12^, ^13^ requires gastroenterologists treating patients with IBD to increasingly consider these complications in their daily care. The study by He therefore is of interest as hypertension constitutes a modifiable risk factor for future cardiovascular disease. However, risk estimates were, while statistically significant, small and the clinical implication of this increased risk remain unclear. Also, whether findings can be transferred to other populations need further confirmation in other populations. Finally, the most effective means of preventing or reducing the burden of most extra‐intestinal diseases is to treat the underlying inflammation of IBD. Whether changes in disease activity state over time influences on the risk of hypertension needs to be determined.

## AUTHOR CONTRIBUTION

Johan Burisch drafted the manuscript.

## CONFLICTS OF INTEREST

Dr. Burisch reports grants and personal fees from AbbVie, grants and personal fees from Janssen‐Cilag, personal fees from Celgene, grants and personal fees from MSD, personal fees from Pfizer, grants and personal fees from Takeda, grants and personal fees from Tillots Pharma, personal fees from Samsung Bioepis, grants and personal fees from Bristol Myers Squibb, grants from Novo Nordisk, personal fees from Pharmacosmos, personal fees from Ferring, personal fees from Galapagos, outside the submitted work.

## Data Availability

Not applicable, this is an invited editorial.

## References

[1] Hedin CRH , Vavricka SR , Stagg AJ , Schoepfer A , Raine T , Puig L , et al. The pathogenesis of extraintestinal manifestations: implications for IBD research, diagnosis, and therapy. J Crohns Colitis. 2019;13(5):541–54. 10.1093/ecco-jcc/jjy191. http://www.ncbi.nlm.nih.gov/pubmed/30445584 30445584

[2] Fiorino G , Lytras T , Younge L , Fidalgo C , Coenen S , Chaparro M , et al. Quality of care standards in inflammatory bowel diseases: a European Crohn’s and colitis organisation [ECCO] position paper. J Crohns Colitis. 2020;14(8):1037–48. https://academic.oup.com/ecco‐jcc/advance‐article/doi/10.1093/ecco‐jcc/jjaa023/5730297 32032423 10.1093/ecco-jcc/jjaa023

[3] Conrad N , Verbeke G , Molenberghs G , Goetschalckx L , Callender T , Cambridge G , et al. Autoimmune diseases and cardiovascular risk: a population‐based study on 19 autoimmune diseases and 12 cardiovascular diseases in 22 million individuals in the UK. Lancet. 2022;400(10354):733–43. 10.1016/S0140-6736(22)01349-6 36041475

[4] Kirchgesner J , Beaugerie L , Carrat F , Andersen NN , Jess T , Schwarzinger M . Increased risk of acute arterial events in young patients and severely active IBD: a nationwide French cohort study. Gut. 2018;67(7):1261–8. 10.1136/gutjnl-2017-314015. http://www.ncbi.nlm.nih.gov/pubmed/28647686 28647686

[5] Ridker PM , Everett BM , Thuren T , MacFadyen JG , Chang WH , Ballantyne C , et al. Antiinflammatory therapy with canakinumab for atherosclerotic disease. N Engl J Med. 2017;377(12):1119–31. 10.1056/nejmoa1707914. http://www.ncbi.nlm.nih.gov/pubmed/28845751 28845751

[6] Hansson GK . Inflammation, atherosclerosis, and coronary artery disease. N Engl J Med. 2005;352(16):1685–95. 10.1056/nejmra043430. http://www.ncbi.nlm.nih.gov/pubmed/15843671 15843671

[7] Panoulas VF , Douglas KMJ , Milionis HJ , Stavropoulos‐Kalinglou A , Nightingale P , Kita MD , et al. Prevalence and associations of hypertension and its control in patients with rheumatoid arthritis. Rheumatol. 2007;46(9):1477–82. 10.1093/rheumatology/kem169. http://www.ncbi.nlm.nih.gov/pubmed/17704521 17704521

[8] Neimann AL , Shin DB , Wang X , Margolis DJ , Troxel AB , Gelfand JM . Prevalence of cardiovascular risk factors in patients with psoriasis. J Am Acad Dermatol. 2006;55(5):829–35. 10.1016/j.jaad.2006.08.040. http://www.ncbi.nlm.nih.gov/pubmed/17052489 17052489

[9] Solak Y , Afsar B , Vaziri ND , Aslan G , Yalcin CE , Covic A , et al. Hypertension as an autoimmune and inflammatory disease. Hypertens Res. 2016;39(8):567–73. 10.1038/hr.2016.35. http://www.ncbi.nlm.nih.gov/pubmed/27053010 27053010

[10] He J , Zhang S , Qiu Y , Liu F , Liu Z , Liu Z , et al. Ulcerative colitis increases risk of hypertension in a UK biobank cohort study. United Eur Gastroenterol J. 2022. http://www.ncbi.nlm.nih.gov/pubmed/36507867 10.1002/ueg2.12351 PMC9892434 36507867

[11] Kaplan GG , Windsor JW . The four epidemiological stages in the global evolution of inflammatory bowel disease. Nat Rev Gastroenterol Hepatol. 2021;18(1):56–66. 10.1038/s41575-020-00360-x 33033392 PMC7542092

[12] Solitano V , Fiorino G , D’Amico F , Peyrin‐Biroulet L , Danese S . Thrombosis in IBD in the era of JAK inhibition. Curr Drug Targets. 2021;22(1):126–36. 10.2174/1389450121666200902164240. http://www.ncbi.nlm.nih.gov/pubmed/32881668 32881668

[13] Sandborn WJ , Feagan BG , D’Haens G , Wolf DC , Jovanovic I , Hanauer SB , et al. Ozanimod as induction and maintenance therapy for ulcerative colitis. N Engl J Med. 2021;385(14):1280–91. 10.1056/nejmoa2033617. http://www.ncbi.nlm.nih.gov/pubmed/34587385 34587385

